# Tailoring Copper Pillars to Prevent Physical Aging in Matrimid^®^ 5218 Carbon Molecular Sieve Membranes

**DOI:** 10.3390/membranes16040133

**Published:** 2026-04-01

**Authors:** Whitney K. Cosey, Edson V. Perez, Kenneth J. Balkus, John P. Ferraris, Inga H. Musselman

**Affiliations:** Department of Chemistry and Biochemistry, The University of Texas at Dallas, Richardson, TX 75080, USA

**Keywords:** CMSM, membrane aging, gas separation, carbon membrane, CMSM pillaring

## Abstract

Carbon molecular sieve membranes (CMSMs) provide a means to greatly improve gas separations. CMSMs have a pore size distribution comprising micropores and ultramicropores which provide high flux and high selectivity, enabling them to outperform polymeric membranes. CMSMs, however, suffer from physical aging that results from the collapse of the pores that severely reduces their gas permeability. Therefore, reducing physical aging of CMSMs is an important step in the development of these types of materials. In this work, a method for reducing physical aging through the incorporation of metal nanoparticles that serve as a structural scaffold for the membrane pore structure is presented. The pore structure in CMSMs is dependent upon the polymeric precursor, and thus the support system incorporated must be tailored. The copper nanoparticles were formed in situ from soluble, copper-based metal–organic polyhedra 18 (MOP-18) dispersed into Matrimid^®^ 5218, a low free volume polymer. The size (2 to 20 nm) and shape (sphere, rods) of the copper particles were refined by adjusting MOP-18 loading, pyrolysis temperature, and soaking time. The Cu-pillared Matrimid^®^ 5218 CMSMs from this work showed no decline in permeability or selectivity for methane (107 Barrer) and carbon dioxide (1785 Barrer) over a period of 21 d. The results suggest that tailored metal pillars can suppress physical aging in CMSMs, thereby enhancing their long-term stability and applicability in gas separations.

## 1. Introduction

The highly developed internal surface area and porosity of carbon molecular sieve membranes (CMSMs) make them excellent membrane materials to surpass the performance of polymeric membranes for gas separations [1,2,3,4,5,6,7]. CMSMs are composed of ultramicropores and micropores, providing the membrane with high selectivity and high permeability. While carbon membranes are known for enhanced permselectivity and high chemical and thermal stability compared to their precursors, they have a flaw that prevents their large-scale utilization: chemical and physical aging [8,9,10,11,12,13,14,15]. Chemical aging, the chemisorption and physisorption of unwanted species, affects the membrane’s permeability. Physical aging, the collapse of a membrane’s inherent pore structure, results in the densification of the membrane. Physical aging is drawing much attention because methods for its reduction, prevention, or regeneration of porosity have not been fully developed. Thus, preparing carbon materials with a strictly controlled and stable pore structure is of great interest.

Physical aging within CMSMs is driven by two factors. The first is noncovalent forces, such as π–π interactions, which are most significant for large graphitic domains. The thermodynamic driving force of graphitic domains to restack leads to pore collapse. The second factor results from the carbonization of high free volume polymer precursors, which yields smaller graphitic domains with a higher density of sp^3^ carbons. The open structures of these carbons are not fully connected and collapse over time [10,11]. Almost all efforts to control microporosity in carbon membranes have been directed toward altering the final pyrolysis temperature [16,17,18]. Researchers have observed that increasing the final pyrolysis temperature narrows the pore structure, which may play a role in minimizing the effects of physical aging. However, this approach tends to yield membranes that are excessively brittle, impeding their applicability for industrial use. Other methods explored to prevent aging involved cross-linking of the polymer precursor to induce the formation of more stable graphitic domains [19], doping the polymer precursor with metals before pyrolysis to retain the pore structure [20], introduction of silicon in the CMSM from the incorporation of siloxane in the polymer precursor [21], and metal pillaring of CMSMs derived from high free volume polymers [22,23]. These methods have shown to be promising in preventing aging but are not always possible to apply to different polymer precursors. For example, chemical or thermal cross-linking of polymers is not always possible without modifications to the polymer structure and properties. The doping of polymers with metals is also a challenge since not all metal carriers are compatible with solvents and polymers, limiting the metal loading in the polymer due to gelation. In one study, the maximum metal loading was limited to 3.2 wt.% due this problem [20]. Incorporation of silicon also faces challenges as the polymer has to be chemically modified to incorporate the silicon precursor. Aging studies from this strategy also showed a significant initial loss of permeability of up to 35% before aging was mitigated [21].

An alternate method to these strategies is to incorporate an additive into the precursor polymer that will remain upon pyrolysis, supporting the pore structure and preventing densification of the membrane. Metal nanoparticles (metal pillaring) can be used to scaffold the microstructure of carbon; their high thermal stability and tunability make them an ideal material to be used in CMSMs [22,23]. A key aspect for the effectiveness of this strategy that has not yet been explored, however, is the tuning of the size of the metal pillars in CMSMs. This capability is needed to generate particles that would fit within the micropores to prevent collapse of the graphitic domains.

Several methods for the synthesis of metal nanoparticles have been reported. In addition, protocols have been developed that focus on the fine tuning of nanoparticle physical and chemical properties, including producing progressively smaller nanoparticle sizes [24,25,26]. Thus, tailoring the size of the metal nanoparticles to preserve a CMSM’s free volume is a plausible method that can be used to minimize physical aging. The synthesis of metal pillars with sizes ranging from 2 to 10 nm would be ideal for supporting low free volume polymer precursors such as Matrimid^®^ 5218 and polybenzimidazole (PBI). On the other hand, high free volume polymer precursors, such as polymer of intrinsic microporosity 1 (PIM-1) [22], BTDA-BAF [23], and 6FDA-DAM, whose resulting CMSM frameworks are composed of large unconnected domains, would benefit from larger 10 to 20 nm pillars.

The literature addresses several experimental parameters in the chemical syntheses of metal nanoparticles, including temperature, concentration of chemicals, and use of capping agents [26], which alters the shape and size distribution of the particles. While these experimental parameters can be monitored and controlled, there is still the need to consider two principles: LaMer’s model of nucleation and Ostwald ripening. In LaMer’s model for particle formation, the metal particles are produced via instantaneous nucleation and their growth is controlled by diffusion [27]. After the formation of a metal nanoparticle, growth is then influenced by sintering and/or Ostwald ripening. This model predicts that smaller nanoparticles would preferentially adsorb onto larger nanoparticles due to their higher surface energy [28]. With increasing time, the size distribution of particles would increase as the larger particles would grow at the expense of the smaller ones [29]. Thus, by minimizing particle–particle encounters, it may be possible to achieve smaller particles as well as a more uniform distribution of particle sizes.

Previous studies focused on the effectiveness metal pillaring had on minimizing aging in CMSMs with great success. These CMSMs, derived from high free volume polymers with different functionalities, showed MOP-18 was a good carrier of copper ions with a good affinity for these polymers [22,23]. Based on these studies, it is clear that metal nanoparticle size and dispersion can be controlled and optimized to accommodate carbon membranes with specific pore size distributions. In this work, the pore structures in CMSMs derived from the low free volume polymer Matrimid^®^ 5218 are pillared with copper nanoparticles, derived from soluble, copper-based, metal–organic polyhedra 18 (MOP-18). MOP-18 was dispersed into Matrimid^®^ 5218 at loadings ranging from 0% (*w*/*w*) to 80% (*w*/*w*), allowing for the in situ formation of copper nanoparticles during pyrolysis [30]. The size (2 to 20 nm) and shape (sphere, rods) of the copper particles are refined by adjusting three parameters: MOP-18 loading, pyrolysis temperature, and soaking time. The best particle size distribution and quantity was achieved with a 40% (*w*/*w*) loading of MOP-18 in Matrimid^®^ 5218. This loading was therefore used to determine the effect the polymer precursor would have on the metal nanoparticle’s distribution and on the gas separation properties of the resulting CMSMs.

## 2. Materials and Methods

### 2.1. Materials

Matrimid^®^ 5218 (Huntsman Holland BV, Rotterdam, The Netherlands) was dried at 120 °C for 1 d in a vacuum oven before use.

MOP-18: Sodium bicarbonate, 1-iodododecane, 5-hydroxyisophthalic acid, copper acetate monohydrate, ethyl acetate, acetonitrile, potassium hydroxide, methanol, 1,1,2,2-tetrachloroethane (TCE), dimethyl formamide (DMF), and chloroform, all with purities greater than 97%, were obtained from Sigma Aldrich, St. Louis, MO, USA. Ethyl ether (99.9%) and hydrochloric acid were purchased from J.T. Baker (Phillipsburg, NJ, USA). Concentrated sulfuric acid was purchased from Mallinckrodt (Paris, KY, USA). Methanol and DMF were dried over 4A activated molecular sieves prior to use [31].

### 2.2. MOP-18 Synthesis

The MOP-18 linker, 5-dodecoxyisophthalic acid, was prepared by esterifying 5-hydroxyisophthalic acid with ethanol. Alkylation of the hydroxyl group of the diester, followed by hydrolysis, yielded 5-dodecoxyisophthalic acid. For the synthesis of MOP-18, separate solutions of the linker and copper acetate in DMF were prepared. The linker (2.7 g, 7.6 mmol) was dissolved in 100 mL of DMF at 80 °C and stirred until a clear solution was obtained. Copper acetate (1.5 g, 7.6 mmol) was dissolved in 50 mL of DMF and stirred at room temperature until fully dissolved. The two solutions were then combined and then 100 mL of methanol was added to precipitate the MOP-18 molecules. The precipitate was allowed to crystallize for 1 d at room temperature, washed twice with methanol, filtered, and dried in a vacuum oven at 60 °C for 1 d to yield blue MOP-18 crystals (93% yield) [31,32,33].

### 2.3. Membrane Preparation

MOP-18 was incorporated into Matrimid^®^ 5218 to form mixed-matrix membranes (MMMs). MMM solutions for membrane casting were prepared by dissolving 0.5 g of Matrimid^®^ 5218 in 4 g of TCE, and by dissolving 0.2 g of MOP-18 in 3 g of TCE. The solutions were then stirred for 1 d and then combined. The 40% (*w*/*w*) MOP-18/Matrimid^®^ 5218 clear blue/green solution was then stirred for 1 d [31]. Pristine membranes were prepared by dissolving 0.5 g of Matrimid^®^ 5218 in 3 g of TCE and stirring the solution for 1 d at room temperature. Before casting, the pure polymer and the MMM solutions were concentrated by evaporating excess solvent until the solid’s concentration reached 12 wt%. A Sheen 1133N (Sheen Instruments Ltd., Kingston, UK) automatic applicator with a doctor blade in a laminar flow hood was then used to cast the membranes on a glass substrate. After drying at room temperature for 2 d, the membranes were removed from the substrate and then annealed in a vacuum oven at 150 °C for 1 d. The average membrane thickness for MMMs and the pure polymer was 80 μm.

CMSMs were formed by pyrolyzing flat polymer membrane precursors. A 3-zone tube furnace (MTI OTF-1200X-III, Richmond, CA, USA) with a quartz tube was fitted with a PID temperature controller (Omega Engineering, Inc., model CN1507TC, Norwalk, CT, USA). Precursor membranes were placed on a graphite plate inside the quartz tube. Pyrolysis under a continuous flow of ultra-high purity nitrogen (UHP, 200 cm^3^/min) was carried out following the protocols shown in Table 1. The CMSMs were removed from the furnace after cooling to room temperature under nitrogen. Labeling of the CMSMs in this work is simplified by appending the final pyrolysis temperature (°C) and soak time (h). For example, a membrane pyrolyzed at 550 °C and soaked for 2 h is written as 550-2.

### 2.4. Characterization

A JEOL 1400+ (Peabody, MA, USA) transmission electron microscope (TEM) operating at 200 kV was used to image the copper particles present within the CMSM. For TEM imaging, a sample of a MOP-18/Matrimid^®^ 5218 MMM at each MOP-18 loading was pyrolyzed at the various conditions listed in Table 1. Samples for TEM were prepared by micronizing the flat carbon membranes using a Resodyn LabRAM Acoustic Mixer (Butte, MT, USA) at 30 G for 30 min. The powder was then dispersed in methanol from which samples were collected using stainless steel grids (200 mesh, Ted Pella, Redding, CA,USA). The sizes of 200 to 1000 copper particles observed in the TEM images were quantified using ImageJ 1.5.

Raman spectra of the CMSMs were obtained using a Thermo Scientific DXR Raman spectrophotometer (Waltham, MA, USA) with a 532 nm laser. X-ray diffractograms of CMSMs as well as of MOP-18 were obtained using a Rigaku Ultima III diffractometer (Woodlands, TX, USA) with Cu K_α_ X-ray radiation.

Thermal gravimetric analysis (TGA) was performed using a PerkinElmer Pyris 1 TGA thermogravimetric analyzer (Shelton, CT, USA) under a UHP nitrogen flow of 20 cm^3^/min and a ramp rate of 10 °C/min. A TA instruments differential scanning calorimeter DSC Q200 (New Castle, DE, USA) was used to measure heat flow during thermal transitions.

Carbon dioxide and nitrogen gas adsorption isotherms were obtained using a Micromeritics ASAP 2020 plus gas adsorption analyzer (Norcross, GA, USA). The CMSMs pore size distribution was calculated using the 2D-NLDFT method implemented in the SAIEUS 3.0 software.

### 2.5. Pure Gas Permeation Measurements

Pure gas permeability of the membranes was measured using custom-built permeameters with LabVIEW 16.0 software interface (National Instruments, Austin, TX, USA) [34]. Flat membranes with known areas and thicknesses were mounted into permeation cells and degassed for 12 h before testing at 35 °C and 2 atm with carbon dioxide or methane. A gas leak test was also performed to ensure accurate permeabilities were obtained. Equation (1) was used to calculate the membrane’s permeability (*P_i_*), utilizing the flux of the gas (*n_i_*), the membrane thickness (*l*), and the transmembrane pressure difference (Δ*p_i_*) [35]. Ideal gas selectivity was calculated as the ratio of the permeabilities of carbon dioxide and methane.

(1) $$P_{i}\;=\frac{n_{i}\;l}{{∆p}_{i}}\;\;,$$

### 2.6. Gas Mixture Permeation Measurements

The custom built permeameters [34], in conjunction with an SRI-3610 gas chromatograph equipped with a thermal conductivity detector, were used for mixed gas separation experiments using an equimolar CO_2_/CH_4_ blend. Calibration of the gas chromatograph was carried out by injecting pure carbon dioxide and methane gas samples into the sampling loop at pressures between 20 Torr and 760 Torr. The detector signal intensity versus pressure in the sampling loop was plotted to obtain calibration curves. For mixed gas experiments, the cell was maintained at 35 °C with an upstream pressure of 3.0 atm. A stage cut (*Q*) of 0.1 was used to minimize concentration polarization at the surface of the membrane (Equation (2)). The mixed gas flow rate across the membrane (*Q_P_*) was first obtained from the gas mixture permeability data without a stage cut. Then with the stage cut set to 0.1 and using *Q_P_*, the retentate purge rate (*Q_R_*) was calculated according to Equation (2) [34]. Experimentally, the retentate purge rate was controlled by a mass flow controller set to the calculated *Q_R_* flow rate connected to the feed side.

(2) $$Q\;=\frac{\left(Q_{P}\right)}{\left(Q_{P}+Q_{R}\right)}\;\;,$$

For each gas mixture experiment, the permeate in the downstream side was evacuated once the flow rate reached steady state. Upon obtaining sufficient pressure in the downstream side (>600 Torr), the permeate was injected into the gas chromatograph sampling loop at 500 Torr. The mixed gas selectivity was determined by calculating the ratio of the detector signals for each gas using their calibration curves. The permeabilities for carbon dioxide and methane were then back calculated from the permeability experiment.

### 2.7. Aging Under Pressure

The apparatus described in the previous section was used to measure membrane aging in pristine polymer-derived CMSMs and Cu-pillared CMSMs. The membranes remained in the permeation cell for the entire duration of the 21 d experiments. Aging in the presence of carbon dioxide and of methane was performed by first evacuating the upstream and downstream sides of the membranes for 1 d. Before each run of the same gas, and when switching to a different gas, the upstream and downstream sides were degassed for 4 h. The gas permeation experiments were carried out at 35 °C and 2 atm (pure gases) or 3 atm (gas mixture).

## 3. Results

### 3.1. Thermal Analysis and Spectroscopy

**Figure 1:**
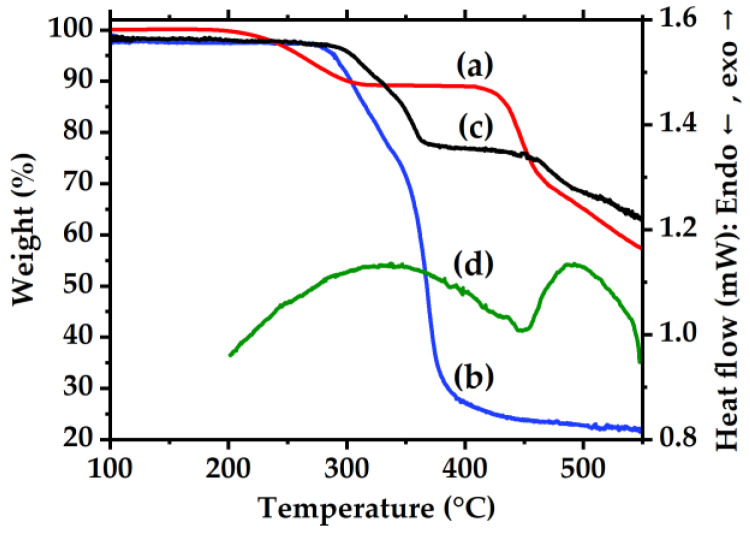
TGA under nitrogen of (a) pristine Matrimid^®^ 5218, (b) MOP-18, (c) 40% (*w*/*w*) MOP-18/Matrimid^®^ 5218 MMM, and (d) DSC plot of the second thermal cycle of the 40% (*w*/*w*) MOP-18/Matrimid^®^ 5218 MMM after the first thermal cycle to 550-2.

Figure 1a–c show TGA curves of pristine Matrimid^®^ 5218, MOP-18, and a 40% (*w*/*w*) MOP-18/Matrimid^®^ 5218 MMM, respectively. The membranes exhibit up to a 40% weight loss at 450 °C due to the decomposition of the polymer and a 60% remaining weight at 550 °C, attributed to the formation of the CMSMs. The TGA of MOP-18 also shows a large weight loss that starts at 300 °C and continues up to 400 °C where the remaining weight is 20%. The MMM also shows a small weight loss that starts at 300 °C due to the decomposition of MOP-18.

Figure 1d shows a depression in heat flow at 450 °C that is attributed to the melting temperature for the copper particles present within the CMSMs after the first thermal cycle to 550-2. It should be noted though that despite the melting temperature of the copper particles is at the same temperature of the polymer decomposition, in the second DSC thermal cycle, the phase change in the copper particles is only observed since the polymer decomposed in the first thermal cycle. A depression in melting temperature is a common phenomenon observed for nanoparticles compared to that of the bulk metal [26,36,37]. It has also been theoretically and experimentally determined that particles smaller than 20 nm can exhibit a phase transition from solid to liquid at temperatures less than 550 °C [36,37]. This phenomenon plays an important role in the ability of nanoparticles to migrate and agglomerate within the CMSM at the carbonization temperatures used in this work. The migration of nanoparticles in the liquid state within the membrane is described by LaMer’s model [27], where the growth of metal particles is driven by diffusion. However, upon approaching a diameter of 20 nm, particles transition back to the solid state and are then influenced by Ostwald ripening where larger particles grow at the expense of smaller particles in order to reduce the overall energy of the system [28,37]. Factors contributing to particle growth and particle size distribution are discussed further in the imaging section.

**Figure 2:**
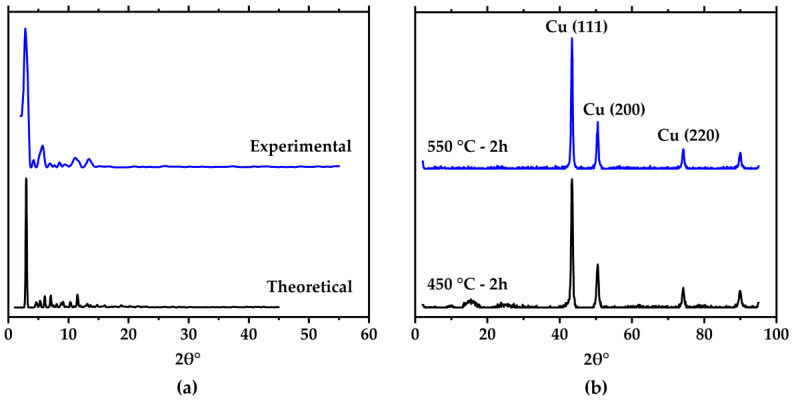
(**a**) MOP-18 experimental (this work) and theoretical XRD patterns calculated from crystallographic data [33]. (**b**) XRD patterns of CMSMs derived from 40% (*w*/*w*) MOP-18/Matrimid^®^ 5218 MMMs pyrolyzed at 450-2h and at 550-2h.

The XRD patterns of the synthesized MOP-18 and those for the 40% (*w*/*w*) MOP-18/Matrimid^®^ 5218 CMSM 450-2 and 550-2 are shown in Figure 2a,b, respectively. The XRD of the synthesized MOP-18, recrystallized from hexanes/octanol, matches well with the theoretical XRD pattern [33], confirming MOP-18 was successfully synthesized. Small variations in the XRD pattern are due to differences in the amounts of octanol present in the pores of recrystallized MOP-18 and in the single crystal from which the structure of MOP-18 and its theoretical XRD pattern was obtained. XRD patterns of the CMSMs (Figure 2b) also support the formation of copper particles in the CMSM at temperatures as low as 450 °C. The 2*θ*° peaks at 43°, 50°, and 74° correspond to the 111, 200, and 220 reflections of copper metal. This confirms that copper nanoparticles are produced in situ upon the thermal degradation of MOP-18 in the MMM.

A copper crystallite size of 20 nm was calculated by applying the Scherrer equation (Equation (3)) to the XRD patterns of the 40% (*w*/*w*) MOP-18/Matrimid^®^ 5218 CMSMs. In Equation (3), *D_p_* is the average crystallite size (nm), *λ* is the X-ray wavelength (Cu-K_α_, 1.54 Å), and *β* is line broadening in radians. Comparing this result to the calculated average crystallite size of 14 nm from MOP-18/PIM-1-derived CMSMs [22], it is apparent that high free volume and low free volume membranes produce small particles with comparable sizes. An insight on the mechanisms for the small variation in particle size (20 nm vs. 14 nm) may come from the Raman spectroscopy of the CMSMs.

(3) $$D_{p}=\frac{(0.94\;\lambda )\;\;}{\left(\beta \;Cos\theta \right)},$$

**Figure 3:**
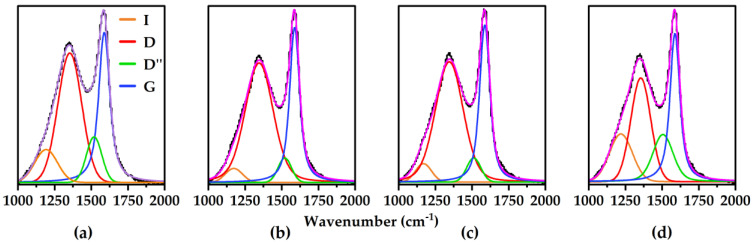
Raman spectra of MOP-18/Matrimid^®^ 5218 CMSMs 550-2 with (**a**) 0% (*w*/*w*), (**b**) 20% (*w*/*w*), (**c**) 40% (*w*/*w*), and (**d**) 80% (*w*/*w*) loadings of MOP-18. Experimental spectrum (**―**) and Raman bands from deconvolution: D-band (**―**), G-band (**―**), I-band (**―**), D”-band (**―**), and intensity obtained from the sum of the deconvoluted bands (**―**).

The mechanisms by which physical aging occurs can be probed by Raman spectroscopy. CMSMs with low *I_D_*/*I_G_* ratios yield large graphitic crystallite sizes with extended conjugation. It is hypothesized that π–π interactions lead to restacking of these graphene-like domains causing pore collapse. On the other hand, CMSMs with high *I_D_*/*I_G_* ratios have small unconnected domains, which lead to a rearrangement of the CMSM with time. In this work, Raman spectroscopy was used to analyze the turbostratic carbon and to determine the effect the 12-C chain of MOP-18 has on the graphitic nature of the CMSMs 550-2. As shown in Figure 3a, the *I_D_*/*I_G_* ratio of pyrolyzed Matrimid^®^ 5218 was determined to be 0.82. However, upon incorporation of MOP-18, the *I_D_*/*I_G_* ratios, calculated from height ratios from deconvoluted spectra shown in Figure 3b–d, decreased to 0.77 at 20% (*w*/*w*) loading, to 0.73 at 40% (*w*/*w*) loading, and to 0.63 at 80% (*w*/*w*) loading suggesting MOP-18 is contributing to the formation of graphitic domains. It was reported that the pyrolysis of MOP-18 alone yields an *I_D_*/*I_G_* ratio of 0.72 [22] which, in the case of this work, may alter the carbon content of the MOP-18/Matrimid^®^ 5218-derived CMSMs and their corresponding *I_D_*/*I_G_* ratios. For example, an *I_D_*/*I_G_* ratio decline of 0.09 from the pure polymer-derived CMSM 550-2 is observed for the 40% (*w*/*w*) MOP-18/Matrimid CMSM 550-2. This shift in Raman intensity to a lower *I_D_*/*I_G_* ratio is a trend that holds for PIM-1-derived CMSMs as well [22] where a decline in the *I_D_*/*I_G_* ratio of 0.12 was also observed (Table 2).

The change in the *I_D_*/*I_G_* ratio is of importance because the value can be used to characterize the carbon and to calculate the graphitic crystallite size *L_a_* (Å) using Equation (4). Since the degree of sp^2^ clustering is proportional to the crystallite size, a decrease in *L_a_* increases the D band intensity and shifts the G band to lower wavelengths as seen in Table 2 for both the 550-2 and 900-2 CMSMs. Extended conjugation within CMSMs corresponds to a low *I_D_*/*I_G_* ratio and is a signature of having large crystallite sizes [38]. An interesting observation from Table 2 is the trend *L_a_* follows with polymer free volume. As the polymer’s free volume increases (BTDA-BAF > PIM-1 > Matrimid^®^ 5218), the *L_a_* decreases in both the pure polymer-derived CMSMs and the pillared CMSMs. This could probably be due to the inability of molecular fragments to grow rapidly during pyrolysis due to the lack of proximity to other fragments. In all these cases, however, the CMSMs derived from MOP-18-containing polymers exhibit larger *L_a_* values than the pure polymers.

(4) $$L_{a}=44{\left(\frac{I_{D}}{I_{G}}\right)}^{-1}$$

From Table 2, it can be extrapolated that the incorporation of MOP-18 increases the average crystallite size, and may thus be a factor in its ability to minimize physical aging. The increase in the pyrolysis temperature from 550 °C to 900 °C for the Matrimid^®^ 5218 system produces CMSMs exhibiting higher *I_D_*/*I_G_* ratios and thus smaller crystallite sizes. This decrease in crystallite size from 6.2 nm to 4.0 nm occurs with both systems, Matrimid^®^ 5218 and PIM-1, due to a breakdown of the graphitic sheets. The crystallite size also impacts the distribution and size of the copper particles. Smaller and more disordered graphitic sheets, subject to stacking imperfections, provide additional pathways for agglomeration to occur on a faster time scale. This could yield a more uniform distribution of particles due to fewer particles existing in the liquid phase after reaching 20 nm.

### 3.2. Imaging

**Figure 4:**
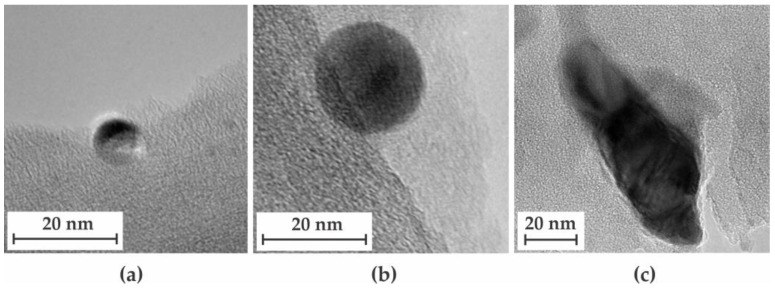
TEM images of CMSMs 550-2 from the pyrolysis of MOP-18/Matrimid^®^ 5218 MMMs containing MOP-18 loadings of: (**a**) 20% (*w*/*w*), (**b**) 40% (*w*/*w*), and (**c**) 80% (*w*/*w*).

**Figure 5:**
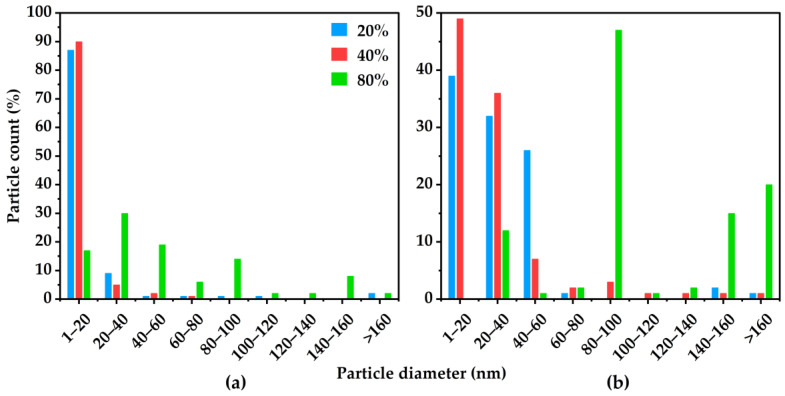
Copper particle size distribution within MOP-18/Matrimid^®^ 5218-derived CMSMs at pyrolysis conditions of (**a**) 550-0 and (**b**) 550-2 and as a function of MOP-18 loadings of 20% (*w*/*w*), 40% (*w*/*w*), and 80% (*w*/*w*).

Transmission electron microscopy was used to image the copper nanoparticles present within the CMSMs. The particles size and distribution were drastically influenced by key factors such as MOP-18 loading, final pyrolysis temperature, and soaking duration. The loading of MOP-18 proved to be key in obtaining copper particles smaller than 20 nm. As shown in Figure 4, membranes pyrolyzed at 550-2 exhibited an increase in the overall size of copper particles with an increase in MOP-18 loading. The 20% (*w*/*w*) loading revealed a high percentage of particles with sizes less than 10 nm (Figure 4a). When the loading was increased from 20% (*w*/*w*) to 40% (*w*/*w*), the average particle size shifted closer to 15 nm, as shown in Figure 4b. The 80% (*w*/*w*) CMSM produced agglomerates that were much too great in size to achieve an even distribution (Figure 4c). Decreasing the soaking duration from 2 h to 0 h allowed for control over the average particle size, by decreasing the time frame in which particle–particle interactions may occur. Membranes pyrolyzed at 550-0 showed a significantly higher number of particles with sizes ranging from 1 to 20 nm (Figure 5a), compared to those pyrolyzed at the same temperature and soaked for 2 h (Figure 5b).

**Figure 6:**
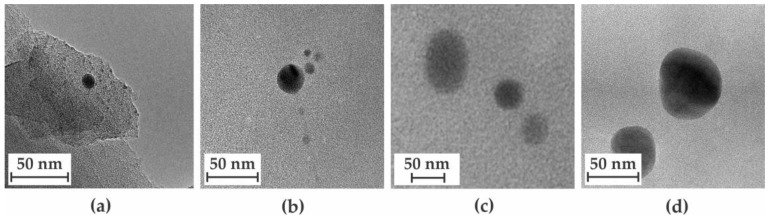
Transmission electron microscopy images of 40% (*w*/*w*) MOP-18/Matrimid^®^ 5218 CMSMs pyrolyzed at (**a**) 550-0, (**b**) 550-2, (**c**) 550-4, and (**d**) 550-6.

**Figure 7:**
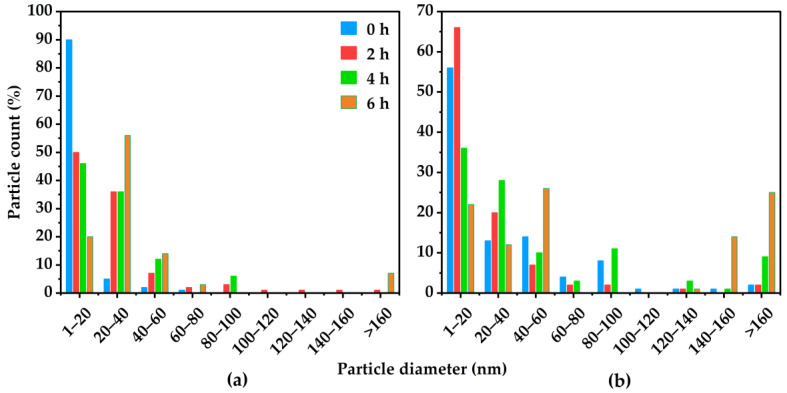
Copper particle size distribution as a function of soaking duration for 0 h, 2 h, 4 h, and 6 h within a 40% (*w*/*w*) MOP-18/Matrimid^®^ 5218-derived CMSM at (**a**) 550 °C and (**b**) 900 °C.

When comparing Figure 5a to Figure 5b, it can be concluded that increasing the soaking time shifts the average particle size to larger values due to an increase in the number of agglomerates. This trend is illustrated in Figure 6, where a 40% (*w*/*w*) MOP-18/Matrimid^®^ 5218 CMSM is soaked at 550 °C from 0 h to 6 h. With an increase in soaking time, more particle–particle interactions occur, resulting in particles with sizes from <10 nm (0 h), 10–20 nm (2 h), 50 nm (4 h), and 60 nm (6 h), as shown in Figure 6a–d and in their corresponding histograms in Figure 7a for CMSMs pyrolyzed at 550 °C, and in Figure 7b for CMSMs pyrolyzed at 900 °C. The histograms also reveal that the size of the majority of the particles lie within the 1 nm to 40 nm range for the CMSMs pyrolyzed at 550 °C, whereas the CMSMs pyrolyzed at 900 °C exhibit a broader particle size distribution. The tapering off of particles after 20 nm is due to the inability of the larger particles to move throughout the membrane. The decline in the number of particles existing in the liquid phase minimizes the growth of particles larger than 20 nm, as shown in the histograms. The particles greater than 20 nm in size are the result of Ostwald ripening [37].

**Figure 8:**
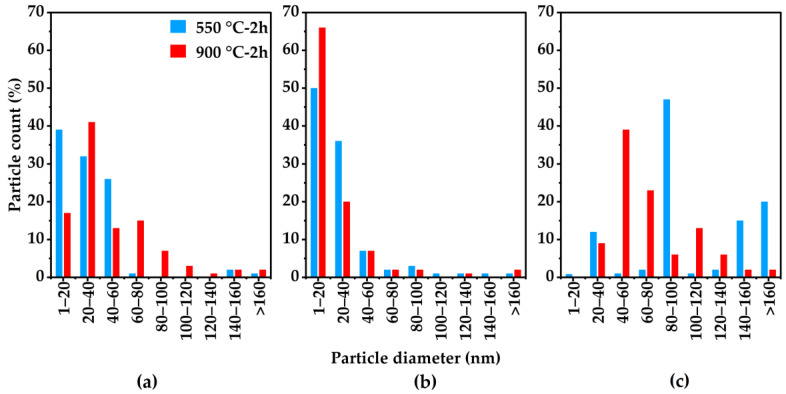
Copper particle size distribution within MOP-18/Matrimid^®^ 5218-derived CMSMs as a function of carbonization temperatures of 550-2 and 900-2 for: (**a**) 20% (*w*/*w*), (**b**) 40% (*w*/*w*), and (**c**) 80% (*w*/*w*) MOP-18 loadings.

Particle size as a function of carbonization temperature and MOP-18 loading was also investigated. When comparing the histograms presented in Figure 8 for each weight loading, the particle size distribution increased slightly for the 550-2 protocol over the 900-2 protocol. An interesting observation is that at 40% loading (Figure 8b), the particle size distribution is narrow, with the particle size staying mostly within 1–20 nm and 20 to 40 nm. The 20% and 80% loadings show, however, a broader size distribution, especially at the 80% loading where the size distribution for the CMSM pyrolyzed at 550 °C differs significantly to that of the CMSM pyrolyzed at 900 °C (Figure 8a,c). This is due to the membrane becoming denser at elevated temperatures, which restricts the particles movement within the CMSM.

Based on these observations, the pyrolysis temperature to achieve small (<20 nm) uniform particles is 550 °C. This pyrolysis temperature minimizes the distortions within the resultant graphitic membrane (fewer sp^3^ terminal carbons), while also minimizing the time allowed for agglomeration to occur. To maximize the effect pillaring would have on the CMSM, copper particles capable of fitting into the pore structure would be most advantageous in order to support the membrane rather than introduce voids. Based on the TEM images presented in Figure 6a,b and the particle size distribution in Figure 7a, the 550-0 and the 550-2 carbonization procedures yield the smallest particles with a uniform size distribution. In addition to these parameters, a high net copper nanoparticle loading is desired to pillar as much of the CMSM material as possible. Since only 15.4% of the molecular weight of MOP-18 corresponds to copper, the MOP-18/Matrimid^®^ 5218 MMM has to have the maximum MOP-18 loading possible without introducing defects. According to Perez et al. [31], a 40 wt.% MOP-18 loading produces MMMs with no gross defects and MOP-18 molecules well dispersed in the matrix. Under these conditions, a maximum of 6% copper is calculated to be present in the CMSMs after pyrolysis. These experimental parameters therefore set the conditions for the best attainable copper nanoparticle loading, size, and dispersion in MOP-18/Matrimid^®^ 5218-derived CMSMs.

**Figure 9:**
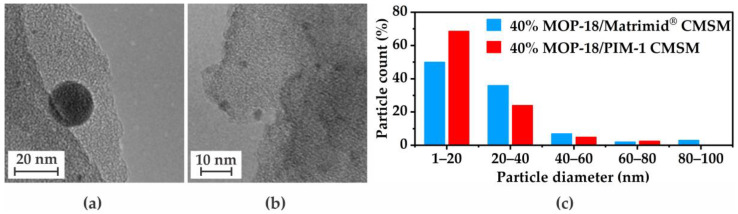
TEM images of (**a**) 40% (*w*/*w*) MOP-18/Matrimid^®^ 5218 CMSM 550-2, (**b**) 40% (*w*/*w*) MOP-18/PIM-1 CMSM 550-2 (adapted with permission from [22]. Copyright 2025 American Chemical Society), and (**c**) their corresponding particle size histograms.

With the data obtained from this work and from the work on PIM-1-derived CMSMs from Cosey et al. [22], a comparison of the effect the free volume of the precursor would have on the particle size can be made. Since the diffusion of particles is a primary factor involved in their agglomeration, the free volume of the precursor may influence the distribution of the resulting particle size upon pyrolysis. The TEM images and particle size distribution for both a 40% (*w*/*w*) MOP-18/Matrimid^®^ 5218 CMSM 500-2 and a 40% (*w*/*w*) MOP-18/PIM-1 CMSM 550-2 are shown in Figure 9a–c. The size distribution obtained suggests that the higher the free volume, the smaller the particles are. This data supports the values calculated by applying the Scherrer equation and is due to the rate at which particles agglomerate. High free volume polymer precursors produce small graphitic sheets upon pyrolysis (*L_a_*, Table 2) that produce both distortions in the form of sp^3^ terminal sites and additional pathways in the form of mesopores. These pathways facilitate particle–particle interaction, and thus occurs more frequently in high free volume systems in comparison to low free volume systems. These interactions lead to particles reaching a size >20 nm in a smaller time frame, which prevents the solid to liquid phase transition of the metal. This transition back to solid minimizes the formation of large agglomerates.

### 3.3. Gas Adsorption

**Figure 10:**
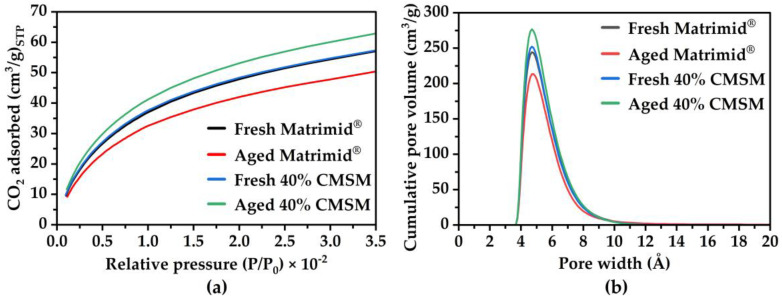
(**a**) Plot of carbon dioxide adsorption at 0 °C and (**b**) pore size distribution from 2D-NLDFT for CMSMs derived from Matrimid^®^ 5218 and 40% (*w*/*w*) MOP-18/Matrimid^®^ 5218 membranes.

The collapse of the pore structure of a membrane, due to physical aging, is a thermodynamically driven process which leads to a dense membrane. Measuring the pore size distribution of Matrimid^®^ 5218-derived CMSMs before and after aging can illustrate the changes that occur due to physical aging. The effect of the incorporation of copper pillars, which aim to prevent the collapse of the micropore structure of the membranes, may also be investigated by measuring the pore size of a copper-pillared CMSM before and after aging. Figure 10a shows the Matrimid^®^ 5218-derived CMSMs carbon dioxide isotherms along with their corresponding pore size distributions in Figure 10b. The measured BET surface area for the fresh Matrimid^®^ 5218-derived CMSM was 268 m^2^/g and 239 m^2^/g for the aged CMSM. Calculated pore volumes were 241 cm^3^/g for the fresh CMSM and 214 cm^3^/g for the aged CMSM, an 11% decrease in pore volume. For the 40% (*w*/*w*) MOP-18/Matrimid^®^ 5218-derived CMSM, the BET surface areas were 278 m^2^/g for the fresh membrane and 292 m^2^/g for the aged membrane. Their corresponding calculated pore volumes increased by 7% from 259 cm^3^/g for the fresh CMSM to 277 cm^3^/g for the aged CMSM. A decline in carbon dioxide adsorption and in the number of ultramicropores is observed for the pristine Matrimid^®^ 5218-derived CMSM as the membrane ages. The data suggest that the rather large graphitic crystallites present in Matrimid^®^ 5218, in comparison to PIM-1 [22], are shifting with time due to π–π interactions which drive the collapse of the ultramicropores in the membrane. The ultramicropores are a result of defects between the graphitic sheets, thus as the CMSM transitions to a more thermodynamically stable structure, the ultramicropores in the membrane are negatively impacted. The copper-pillared fresh Matrimid^®^ 5218 CMSM on the other hand, revealed a slight increase in carbon dioxide adsorption and the number of ultramicropores. The results highlight that the pore structure of the copper-pillared Matrimid^®^ 5218 membranes remains intact, and that the incorporation of pillars prevented a shift in the CMSMs graphitic sheets. The increase in carbon dioxide adsorption and in the number of ultramicropores in the membrane is hypothesized to occur due to the copper pillars creating/directing micropores within the CMSM during the pyrolysis from which a small percentage collapse, generating ultramicropores.

### 3.4. Gas Permeation

**Figure 11:**
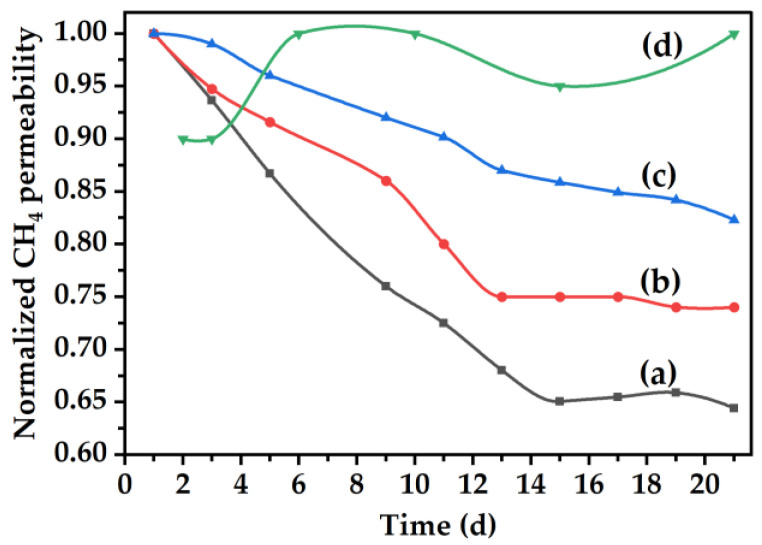
Changes in CH_4_ permeability (pure gas) upon aging at 35 °C and 2 atm for: (a) 0% (*w*/*w*), (b) 20% (*w*/*w*), and (c) 40% (*w*/*w*) MOP-18/Matrimid^®^ 5218 CMSMs 550-2 and (d) aging at 35 °C and 3 atm (gas mixture) for a 40% (*w*/*w*) MOP-18/Matrimid^®^ 5218 CMSMs 550-0.

Characterized by a decline in permeability and increase in selectivity due to the collapse of pore structure [39,40,41,42], physical aging is one of the factors that impedes the industrial application of CMSMs [8,9]. Table 3 shows the pure gas permeabilities and selectivities of fresh and 21 d-aged carbon membranes from this work. Average permeability and standard deviations were determined from the permeabilities obtained using two separate cast membranes for each case. The permeabilities of the aged CMSM illustrate the impact that the loading of MOP-18 and soaking duration have on the membranes effectiveness at reducing physical aging. As it can be observed in Table 3, a 0 h soaking was more effective in minimizing aging than a 2 h soaking for a MOP-18/Matrimid^®^ CMSM since the permeabilities for both carbon dioxide (1785 Barrer) and methane (107 Barrer) remained constant during the 21 d testing period. The gas permeability plot in Figure 11a shows that the CMSM derived from pristine Matrimid^®^ 5218 550-2 collapses rapidly and plateaus at 14 d with a 35% permeability loss, but, upon incorporation of 20% (*w*/*w*) MOP-18, the aging plateaus at 12 d with 25% reduction in permeability (Figure 11b). The incorporation of 40% (*w*/*w*) MOP-18 (Figure 11c) has a greater impact on physical aging prevention when compared to the 0% (*w*/*w*) and the 20% (*w*/*w*) MOP-18 loading with only 15% of permeability decay at 21 d. The variation in copper size distribution obtained with the 20% (*w*/*w*) and 40% (*w*/*w*) MOP-18/Matrimid^®^ 5218 CMSMs 550-2 also minimally impacted the reproducibility of the permeability of the membranes as shown in the percent standard deviations (Table 3).

This enhanced stability to aging despite the low copper loading of 6% in the CMSM and the increased graphitic domain size *L_a_* that may increase the number of π–π interactions may be due to the positioning of the copper particles in the micropore and mesopore regions. The copper particles positioned at these voids may act as anchors that prevent the slippage of the graphitic domains. This is plausible because much of the copper particle size falls in the range of 1–20 nm (Figure 6a and Figure 7a). It is unknown if the remaining amount of copper nanoparticles with sizes < 1 nm may be enough to intercalate between all the graphitic sheets in the entirety of the CMSM or not.

**Figure 12:**
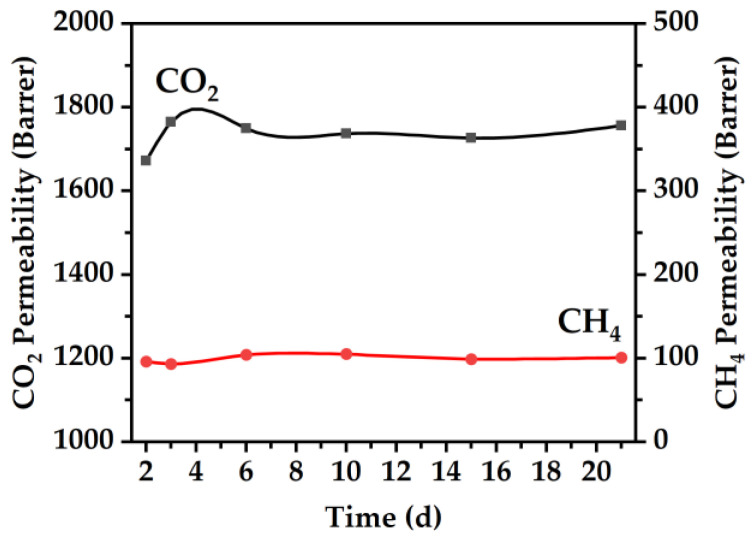
Gas mixture permeability at 35 °C and 3 atm of methane and carbon dioxide for 21 d for a 40% (*w*/*w*) MOP-18/Matrimid^®^ 5218 CMSM 550-0.

By controlling the particle shape and distribution through the carbonization protocol, the ability of the membranes to resist physical aging as well as the reproducibility of the permeability of the membranes is significantly improved. Through shortening the duration at which particles are allowed to interact from 2 to 0 h, a nearly 100% retention in membrane permeability was achieved, as is observed in Figure 11d that shows the normalized methane permeability obtained from gas mixture experiments for a 40% (*w*/*w*) MOP-18/Matrimid^®^ 5218 CMSM 550-0. The CMSM’s resistance to physical aging with gas mixture experiments at 3 atm indicate that pillaring with small nanoparticles is effective. In the case of the 40% (*w*/*w*) MOP-18/Matrimid^®^ 5218 CMSM 550-0, both methane and carbon dioxide retained their initial permeability values of 101 Barrer and 1756 Barrer, respectively, over a period of 21 d as seen in Figure 12.

Though the aging testing period lasted 21 d, this time frame provided important insights on the performance of the copper pillars in minimizing aging in CMSM from both single gas and gas mixture permeation experiments. As the objective of the present study was to determine the effectiveness of the copper pillars in suppressing aging relative to the non-pillared CMSM, the results show to be promising and encouraging for future long-term CMSM aging studies. This is seen in Figure 11 where a fast decline in gas permeation for the non-pillared CMSM is observed within the first 14 d of testing (Figure 11a), whereas no decline in gas permeation is observed for the pillared CMSM during the same time period and up until 21 d (Figure 11d). A similar phenomenon was also observed for other CMSMs where a rapid decline in permeability was observed during the first 14 days of testing and then a slower decline thereafter [43,44]. From these observations, it is then plausible to infer that the rate of aging of the pillared CMSMs could be very slow, which is encouraging, but to assess the full potential and robustness of this method of aging suppression, long-term gas permeation studies with gas mixtures under real-world conditions (e.g., feed pressure, composition, temperature, humidity) may need to be performed.

The incorporation of copper pillars from the incorporation of MOP-18 to reduce physical aging was proven to be effective in Matrimid^®^ 5218 and PIM-1 systems. The solubility of MOP-18 in casting solvents and its affinity with the polymers may have contributed to an even distribution of the copper nanoparticles during pyrolysis that translated into a more effective pillaring. As Cosey et al. also demonstrated, the incorporation of copper pillars from the incorporation of MOP-18 into PIM-1 also allowed for a complete retention in carbon dioxide permeability (4187 Barrers) with only a 20% decrease in methane permeability (252 to 200 Barrers) [22]. The results of the present work also indicate that for the low free volume polymer precursor Matrimid^®^ 5218, a no soaking time is more effective in producing copper pillars for minimizing aging than a 2 h soaking time. This is opposite to the case of the high free volume polymer PIM-1 that is best pillared with copper pillars following a 2 h soaking time.

## 4. Conclusions

The pyrolysis of the MMMs derived from MOP-18 yielded copper nanoparticles in situ; their size and distribution were tailored by varying the loading of MOP-18, the carbonization temperature, and the soaking duration. DSC suggests the melting temperature of the copper nanoparticles to be 450 °C. The depression in melting temperature in comparison to the bulk melting temperature for copper metal (1083 °C) allowed for the agglomeration of metal particles. To minimize particle size and tighten the size distribution, it was determined that for low free volume polymer precursors such as Matrimid^®^ 5218, the 550-0 protocol is more effective in reducing aging which is opposite to that for the high free volume PIM-1 that requires a 550-2 protocol. Gas permeation data proved that the use of copper pillars to scaffold the CMSMs inherent pore structure is effective. The ability to reduce physical aging, as observed with the copper-pillared CMSMs, seems not to be controlled by the functionality of the polymer precursor, potentially making this technique available to other polymer systems but testing with more polymer systems may need to be performed. A functionalization of the MOP-18 linker could be performed to improve compatibility with other polymer systems or solvents if incompatibilities arise.

## Figures and Tables

**Table 1:** Pyrolysis protocols for carbonizations at 550 °C and 900 °C.

Protocol 1	Protocol 2
1. 20–250 °C at a rate of 15 °C/min	1. 20–250 °C at a rate of 15 °C/min
2. 250–535 °C at a rate of 3.85 °C/min	2. 250–535 °C at a rate of 3.85 °C/min
3. 535–550 °C at a rate of 0.25 °C/min	3. 535–550 °C at a rate of 0.25 °C/min
4. 0 h, 2 h, 4 h, or 6 h soak at 550 °C	4. 550–875 °C at a rate of 3.85 °C/min
	5. 875–900 °C at a rate of 0.25 °C/min
	6. 0 h, 2 h, 4 h, or 6 h soak at 900 °C

**Table 2:** *I_D_*/*I_G_* height ratios of CMSMs pyrolyzed for 2 h at 550 °C and at 900 °C along with their corresponding *L_a_* (Å) values and G-band (cm^−1^) positions.

CMSM Precursor	*I_D_*/*I_G_*	*L_a_*	G-Band	*I_D_*/*I_G_*	*L_a_*	G-Band
	550 °C	550 °C	550 °C	900 °C	900 °C	900 °C
Matrimid^®^ 5218	0.82	54	1588	1.03	43	1586
40% (*w*/*w*) MOP-18/Matrimid^®^ 5218	0.73	62	1590	1.09	40	1589
PIM-1 [22] ^(a)^	0.94	47	1590	1.14	39	1590
40% (*w*/*w*) MOP-18/PIM-1 [22] ^(a)^	0.82	54	1585	1.09	40	1586
BTDA-BAF [23]	1.02	43	1581			
40% (*w*/*w*) MOP-18/BTDA-BAF [23]	0.92	48	1580			

^(a)^ Adapted with permission from [22]. Copyright 2025 American Chemical Society.

**Table 3:** Pure gas permeabilities (in Barrer) at 35 °C and 2 atm of fresh and 21 d-aged Matrimid^®^ 5218 and MOP-18 Matrimid^®^ 5218-derived CMSMs (average of two membranes).

CMSM	Fresh	Fresh	Aged	Aged	% Decline	% Decline
	CH_4_	CO_2_	CH_4_	CO_2_	CH_4_	CO_2_
Matrimid^®^ (550-2)	111 ± 9%	1179 ± 4%	71 ± 1%	912 ± 1%	36	23
20% (*w*/*w*) MOP-18/Matrimid^®^ (550-2)	95 ± 14%	1082 ± 4%	70	900	26	17
40% (*w*/*w*) MOP-18/Matrimid^®^ (550-2)	88 ± 15%	1654 ± 15%	73 ± 2%	1389 ± 2%	17	16
40% (*w*/*w*) MOP-18/Matrimid^®^ (550-0)	107 ± 5%	1785 ± 3%	101 ± 1%	1765 ± 1%	0.05	0.01

## Data Availability

Data are available from the author upon request.

## Abbreviations

The following abbreviations are used in this manuscript:

CMSM	Carbon molecular sieve membrane
MOP-18	Metal–organic polyhedra 18
PBI	Polybenzimidazole
PIM-1	Polymer of intrinsic microporosity 1
TCE	1,1,2,2-tetrachloroethane
DMF	Dimethylformamide
MMM	Mixed-matrix membrane
TEM	Transmission electron microscopy
TGA	Thermogravimetric analysis
DSC	Differential scanning calorimetry
XRD	X-ray diffraction
BET	Brunauer-Emmett-Teller surface area
2D-NLDFT	Two-Dimensional Non-Local Density Functional Theory

## References

[1] Jiang L.Y., Chung T.-S., Rajagopalan R. (2007). Dual-Layer Hollow Carbon Fiber Membranes for Gas Separation Consisting of Carbon and Mixed Matrix Layers. Carbon.

[2] Genduso G., Ogieglo W., Wang Y., Pinnau I. (2024). Carbon Molecular Sieve Gas Separation Materials and Membranes: A Comprehensive Review. J. Membr. Sci..

[3] Li H.-j., Liu Y.-d. (2022). A Review of Polymer-Derived Carbon Molecular Sieve Membranes for Gas Separation. New Carbon Mater..

[4] Hu L., Jiao Y., Zhang G., Guan J., Bui V.T., Lin H. (2026). Polymer-Derived Carbon Molecular Sieves with Tailored Polymodal Pores for Membrane Separations. Prog. Polym. Sci..

[5] Sheng L., Mu Q., Hua K., Deng M., Ren J. (2025). Optimizing the Stabilization Environment of the Carbon Molecular Sieve Membranes Derived from the Porous Hollow Fibers for Gas Separation. Ind. Eng. Chem. Res..

[6] Liu Z., Cao Y., Koros W.J. (2024). Synergistic Tuning of Microstructure and Morphology in Carbon Molecular Sieve Hollow Fibers for Propylene/Propane Separation. Angew. Chem. Int. Ed..

[7] Liu Z., Cao Y., Koros W.J. (2024). Polyimide-Derived Carbon Molecule Sieve Membranes for Gas Separations. Nat. Chem. Eng..

[8] Hägg M.-B., Lie J.A., Lindbråthen A. (2003). Carbon Molecular Sieve Membranes. Ann. N. Y. Acad. Sci..

[9] Xu L., Rungta M., Hessler J., Qiu W., Brayden M., Martinez M., Barbay G., Koros W.J. (2014). Physical Aging in Carbon Molecular Sieve Membranes. Carbon.

[10] Puspasari T., Wang Y., Ghanem B., Hu X., Elahi F., Wehbe N., Pinnau I. (2025). Development of High Performance Pyrolized Polyimide-Based Carbon Molecular Sieves for Enhanced Selectivity of Propylene/Propane Gas Separation. Angew. Chem. Int. Ed..

[11] Kim D., Ryu M.-H., Park A., Kim J.-E., Kim S.-J., Kwon Y., Choi J., Park J. (2025). Carbon Molecular Sieve Membranes Derived from Uv-Irradiated Polyimides for Enhanced Molecular Separation and Physical Aging Resistance. J. Membr. Sci..

[12] Han X., Tang J., Kong R., Xue H., Wang W. (2025). Research Progress of Carbon Molecular Sieve Membranes Suitable for Hydrogen Separation. Microporous Mesoporous Mater..

[13] Liu J., Xue P., Yan X., Qi N., Chen Z., Wang Z., Li N. (2025). In Situ Monitoring of Nonlinear Physical Aging and Anti-Aging in Polymer-Based Separation Membranes. J. Membr. Sci..

[14] Cai M., Chen J., Liu H., Sun L., Wu J., Cui T., Zhang S., Ma X., Min Y. (2025). Unveiling the Mystery: How Tr Precursors Lead to Exceptional Gas Separation Performance in Cmsms. J. Membr. Sci..

[15] Zhao G., Wang K., Fang C., Wang Y., Wang D., Song Z., Lei L., Xu Z. (2024). Precise Molecular Sieving Using Metal-Doped Ultramicroporous Carbon Membranes for H_2_ Separation. AlChE J..

[16] Sedghi A., Farsani R.E., Shokuhfar A. (2008). The Effect of Commercial Polyacrylonitrile Fibers Characterizations on the Produced Carbon Fibers Properties. J. Mater. Process. Technol..

[17] Tanihara N., Shimazaki H., Hirayama Y., Nakanishi S., Yoshinaga T., Kusuki Y. (1999). Gas Permeation Properties of Asymmetric Carbon Hollow Fiber Membranes Prepared from Asymmetric Polyimide Hollow Fiber. J. Membr. Sci..

[18] Yoshimune M., Haraya K. (2010). Flexible Carbon Hollow Fiber Membranes Derived from Sulfonated Poly(Phenylene Oxide). Sep. Purif. Technol..

[19] Karunaweera C., Musselman I.H., Balkus K.J., Ferraris J.P. (2019). Fabrication and Characterization of Aging Resistant Carbon Molecular Sieve Membranes for C_3_ Separation Using High Molecular Weight Crosslinkable Polyimide, 6FDA-DABA. J. Membr. Sci..

[20] Chu Y.-H., Yancey D., Xu L., Martinez M., Brayden M., Koros W. (2018). Iron-Containing Carbon Molecular Sieve Membranes for Advanced Olefin/Paraffin Separations. J. Membr. Sci..

[21] Li H., Zhang Q., Xie Z., Zhao B., Yu Y., Liu Y. (2023). Simultaneously Enhanced Gas Permeability, Selectivity and Aging Stability of Carbon Molecular Sieve Membranes by the Molecule Doping of Silicon. Carbon.

[22] Cosey W.K., Balkus K.J., Ferraris J.P., Musselman I.H. (2021). Reduced Aging in Carbon Molecular Sieve Membranes Derived from PIM-1 and MOP-18. Ind. Eng. Chem. Res..

[23] Tajik M., Bin Haque S.F., Perez E.V., Vizuet J.P., Firouzi H.R., Balkus K.J., Musselman I.H., Ferraris J.P. (2023). Pillared Carbon Membranes Derived from Cardo Polymers. Nanomaterials.

[24] Biçer M., Şişman İ. (2010). Controlled Synthesis of Copper Nano/Microstructures Using Ascorbic Acid in Aqueous Ctab Solution. Powder Technol..

[25] Boita J., Nicolao L., Alves M.C.M., Morais J. (2017). Controlled Growth of Metallic Copper Nanoparticles. New J. Chem..

[26] Mott D., Galkowski J., Wang L., Luo J., Zhong C.-J. (2007). Synthesis of Size-Controlled and Shaped Copper Nanoparticles. Langmuir.

[27] Whitehead C.B., Özkar S., Finke R.G. (2019). Lamer’s 1950 Model for Particle Formation of Instantaneous Nucleation and Diffusion-Controlled Growth: A Historical Look at the Model’s Origins, Assumptions, Equations, and Underlying Sulfur Sol Formation Kinetics Data. Chem. Mater..

[28] Ouyang R., Liu J.-X., Li W.-X. (2013). Atomistic Theory of Ostwald Ripening and Disintegration of Supported Metal Particles under Reaction Conditions. J. Am. Chem. Soc..

[29] Meakin P. (1990). Diffusion-Limited Droplet Coalescence. Phys. A Stat. Mech. Appl..

[30] Cosey W.K. (2020). Prevention of Physical Aging Within Carbon Molecular Sieve Membranes for Gas Separations. Doctoral Dissertation.

[31] Perez E.V., Balkus K.J., Ferraris J.P., Musselman I.H. (2014). Metal-Organic Polyhedra 18 Mixed-Matrix Membranes for Gas Separation. J. Membr. Sci..

[32] Lu Z., Knobler C.B., Furukawa H., Wang B., Liu G., Yaghi O.M. (2009). Synthesis and Structure of Chemically Stable Metal−Organic Polyhedra. J. Am. Chem. Soc..

[33] Furukawa H., Kim J., Plass K.E., Yaghi O.M. (2006). Crystal Structure, Dissolution, and Deposition of a 5 nm Functionalized Metal-Organic Great Rhombicuboctahedron. J. Am. Chem. Soc..

[34] Perez E.V., Kalaw G.J.D., Ferraris J.P., Balkus K.J., Musselman I.H. (2017). Amine-Functionalized (Al) MIL-53/VTEC™ Mixed-Matrix Membranes for H_2_/CO_2_ Mixture Separations at High Pressure and High Temperature. J. Membr. Sci..

[35] Koros W.J., Fleming G.K. (1993). Membrane-Based Gas Separation. J. Membr. Sci..

[36] Ryu J., Kim H.-S., Hahn H.T. (2011). Reactive Sintering of Copper Nanoparticles Using Intense Pulsed Light for Printed Electronics. J. Electron. Mater..

[37] Yeshchenko O.A., Dmitruk I.M., Alexeenko A.A., Dmytruk A.M. (2007). Size-Dependent Melting of Spherical Copper Nanoparticles Embedded in a Silica Matrix. Phys. Rev. B.

[38] Knight D.S., White W.B. (2011). Characterization of Diamond Films by Raman Spectroscopy. J. Mater. Res..

[39] Cheng Y., Wang Z., Zhao D. (2018). Mixed Matrix Membranes for Natural Gas Upgrading: Current Status and Opportunities. Ind. Eng. Chem. Res..

[40] Luo S., Wiegand J.R., Gao P., Doherty C.M., Hill A.J., Guo R. (2016). Molecular Origins of Fast and Selective Gas Transport in Pentiptycene-Containing Polyimide Membranes and Their Physical Aging Behavior. J. Membr. Sci..

[41] Staiger C.L., Pas S.J., Hill A.J., Cornelius C.J. (2008). Gas Separation, Free Volume Distribution, and Physical Aging of a Highly Microporous Spirobisindane Polymer. Chem. Mater..

[42] Wieneke J.U., Staudt C. (2010). Thermal Stability of 6FDA-(co-)Polyimides Containing Carboxylic Acid Groups. Polym. Degrad. Stabil..

[43] Schlosser S., Qiu W., Liu Z., Campbell Z.S., Koros W.J. (2025). Leveraging Molecular Scale Free Volume Generation to Improve Gas Separation Performance of Carbon Molecular Sieve Membranes. J. Membr. Sci..

[44] Deng M., Yan Z., Zhang Z., Zheng J., Jiang W., Yang L., Yao L., Wang K., Tang J., He X. (2026). Ionic Liquid-Mediated Pore Structure Modulating in Polyimide-Derived Carbon Molecular Sieve Membranes for Enhanced Gas Separation Performance. Chem. Eng. Sci..

